# Supplementary material for: Sequencing 4.3 million mutations in wheat promoters to understand and modify gene expression
Source: Proc Natl Acad Sci U S A. 2023 Sep 13;120(38):e2306494120. doi: 10.1073/pnas.2306494120 (PMC10515147; doi:10.1073/pnas.2306494120)
Supplement: Supplementary file 1 — Appendix 01 (PDF) [file pnas.2306494120.sapp.pdf]

PNAS

Supporting Information for

Sequencing 4.3 million mutations in wheat promoters to understand and modify gene expression

Junli Zhang, Hongchun Xiong, Germán F. Burguener, Hans Vasquez-Gross, Qiujie Liu, Juan M. Debernardi, Alina Akhunova, Kimberly Garland-Campbell, Shahryar F. Kianian, Gina Brown-Guedira, Curtis Pozniak, Justin D. Faris, Eduard Akhunov, Jorge Dubcovsky

Corresponding author: Jorge Dubcovsky
Email: jdubcovsky@ucdavis.edu

This PDF file includes:

Supporting Text, Methods S1 to S5
Tables S1 to S11
SI References 1-6

Other supporting materials for this manuscript include the following: None

Supporting Information Text

Method S1. Library preparation and capture.

For the promoter capture, we re-used DNA sequencing libraries generated in our previous exome capture study (1), which included 1,513 M_2 plants from the tetraploid wheat cultivar 'Kronos' mutagenized with Ethyl Methane Sulfonate (EMS). Briefly, genomic DNA libraries were constructed using the High-Throughput Library Preparation kits from KAPA Biosystems, Inc. (Wilmington, MA, USA, catalog number KK8234) with dual-SPRI bead size selection. The libraries were barcoded using NEXTflex-96™ oligos 1-48 (Bioo Scientific, Austin, TX, USA, catalogue number 514105) and amplified by PCR (five cycles). The products were purified with Agencourt AMPure beads (Beckman Coulter, A63881) on the Sciclone G3 platform and were eluted in ultrapure DNase/RNase free distilled water and stored at -80 °C.

To capture the gene promoter regions, we used a previously published assay (2) based on 2-kb regions upstream of 110,790 high-confidence wheat genes annotated in Chinese Spring (CS) RefSeq v1.0. Probes for this assay were ordered from Roche (SeqCap EZ Prime Developer Probes 96 Reaction, catalog number 8247633001). For the captures, we pooled 24 DNA libraries together (125 ng/sample, 3 µg in total) and mixed the DNA with blocking oligos for Illumina adapters and repetitive DNA sequences (developer reagent). The DNA mixture was dried using a speed vacuum centrifuge. Capture hybridization and washes were done according to recommended protocols from Roche SeqCapEZ User Guide 4.2 v7. Briefly, the DNA pellet was dissolved in the buffers provided in the SeqCap EZ Hybridization and Wash Kit (Roche, catalog number 5634253001), denatured at 95 °C for 10 minutes, and hybridized to the SeqCap EZ Prime Developer probes for the wheat promoter regions (2) (Roche, catalog number 8247633001) for 70 h at 48 °C in a thermocycler (lid temperature set to 58 °C). The hybridization reaction was recovered using Dynabeads M-270 streptavidin beads (Invitrogen, 653-06) and washed according to the manufacturer's protocol (Roche, catalog number 5634253001). We pooled captures from 48 sequencing libraries and sequenced them in one lane of Illumina NovaSeq S4 (PE150) at the UC Davis Genome Center.

Method S2. Identification of SNPs using MAPS.

As a genome reference, we used the CS RefSeq v1.0 genome (3) with the improved CS RefSeq v1.1 annotation. For simplicity, and since the genome coordinates from v1.0 and v1.1 are the same, we will refer to the reference as CS RefSeq v1.1. We aligned the reads from the mutant plants to CS RefSeq v1.1 and called SNPs using default mpileup parameters and mapping quality higher than 20. We then used the MAPS pipeline to select bases in the reference covered by at least one read at quality higher than 20 in a minimum number of samples (MinLib parameter = 38). The minimum number of reads carrying the mutation (minimum coverage, henceforth, MC) required to call that mutation was established independently for homozygous (HomMC) and heterozygous (HetMC). We used four levels of stringency, designated from the lowest to the highest as HetMC3/HomMC2, HetMC4/HomMC3, HetMC5/HomMC3, HetMC6/HomMC4. For each library, we used the lowest stringency level that resulted in a %EMS > 98%, and we refer to this method as EMS98%. This threshold was sufficient to reduce the percent of non-EMS transitions (A>G/T>C) below 0.7 % in the worst lines. The adjustment of the stringency level by library resulted in the elimination of more SNPs from the low-quality libraries and the inclusion of more mutations from the good quality libraries relative to the fixed HetMC5/HomMC3 stringency level used in the previous

exome capture. The new strategy significantly increased the number of called mutations while maintaining a low estimated error.

Method S3. Error rate determination

We used four different levels of stringency to call mutations based on the minimum number of reads showing the mutation in heterozygous and homozygous states. The most stringent combination required a heterozygous mutation to be observed in at least six reads and a homozygous mutation to be observed in at least four reads (henceforth, HetMC6/HomMC4). The most liberal criteria required the heterozygous mutation to be observed in at least three reads and a homozygous mutation to be observed in at least two (henceforth, HetMC3/HomMC2). We also used HetMC4/HomMC3 and HetMC5/HomMC3 as intermediate criteria.

The EMS treatment is expected to produce G to A (G>A) or C to T (C>T) mutations (henceforth EMS mutations). At each stringency level, we determined the percent of C to T and G to A mutations, which is designated hereafter as %-EMS. Although we eliminate all the non-EMS mutations from the final database, we cannot eliminate the G>A or C>T errors included among the real mutations induced by the EMS treatment. To estimate the number of these residual errors we used the frequency of reciprocal non-EMS transitions (A>G or T>C SNPs, henceforth, A>G/T>C), which have a similar frequency to the G>A/C>T changes among random mutations (5).

To select a %-EMS threshold, we tested different %-EMS stringency levels and found that ≥ 98% EMS was sufficient to reduce the percent of non-EMS transitions below 0.7 % in the worst lines. We then selected mutations in each line at the first HetMC/HomMC level that had ≥ 98 % EMS mutations. Most of the libraries (940) exceeded the 98%-EMS threshold at the lowest stringency level (HetMC3/HomMC2). Among the other libraries, 389 exceeded the threshold at HetMC4/HomMC3, 120 at HetMC5/HomMC3 and 64 at the highest stringency level (HetMC6/HomMC4). Twenty-two libraries did not reach the 98% EMS threshold even at the highest stringency level (HetMC6/HomMC4) and were eliminated.

Method S4. Variant Effect Prediction.

After creating a VCF file with all the EMS mutations, we predicted the effects and SIFT scores (4) of all mutations using the Variant Effect Predictor (VEP) program (5) from Ensembl tools release 103 in offline mode. Release 50 of the *T. aestivum* VEP cache file was downloaded from Ensembl (ftp://ftp.ensemblgenomes.org/pub/plants/release-50/variation/vep/triticum_aestivum_vep_50_IWGSC.tar.gz). Commands used to install VEP, run the prediction and count the number of different types of mutations were included in the script file 'variant_effects_prediction_for_exome_capture_SNPs.sh' deposited in Zenodo together with the VCF files (doi.org/10.5281/zenodo.7754136).

Method S5. Estimation of the proportion of mutated "EMS-accessible" G residues.

The large number of mutations detected in promoter and exome captures provided a unique opportunity to estimate the saturation of our mutant libraries, which equals the proportion of "accessible" GC nucleotides carrying at least one mutation in the population (henceforth, mutated sites). The number of mutations per site follows an approximate Poisson distribution, with most of the sites lacking mutations

(n0), a large number carrying one mutation (n1) and rapidly decreasing numbers of sites carrying more than one mutation (n2 to n>7). From our data we can count the number of sites that have one or more mutations, but we need to infer n0 from the Poisson distribution. The main parameter of Poisson distribution is the mean (λ = average number of mutations per site). We estimated the value of λ that maximized the adjustment of the predicted number of sites with mutations in one, two or three lines to the observed values. To do that we selected the λ that minimized the normalized differences between the observed and expected data or $(O-E)^2/E$. In this optimization we excluded the sites with mutation in 4 or more lines to minimize the risk of including residual heterogeneity sites since we observed excess mutation relative to the expected from a Poisson distribution.

Once we optimized λ, we used it to estimate n0 and the accessible GC sites as follows:

λ = total EMS mutations / accessible sites

λ = total EMS mutations / (n0 + mutated sites)

λ = (1 x n1 + 2 x n2 + …) / (n0 + (n1 + n2 + …))

so n0 = (total EMS mutations / λ) - (mutated sites)

and EMS-accessible GC sites = n0 + (mutated sites)

and % saturation of the EMS-accessible space = mutated sites / EMS-accessible GC sites.

Supporting Tables

Table S1. Summary of mutations and uniquely mapped EMS mutations at different stringency levels.

	Global error				Lib. adjusted
Stringency	HetMC3/ HomMC2	HetMC4/ HomMC3	HetMC5/ HomMC3	HetMC6/ HomMC4	EMS 98%
Total SNPs	4,657,354	4,120,688	3,784,020	3,391,163	4,345,625
Total EMS mutations *	4,509,446	4,064,952	3,749,732	3,364,268	4,287,361
EMS / kb (all population)	44.0	39.7	36.6	32.9	41.9
EMS / Mb per line	28.7	25.9	23.9	21.4	27.3
Average %-EMS	96.8	98.6	99.1	99.2	98.7
Lowest %-EMS	34.8	72.2	91.5	95.8	98.0
Highest % EMS	99.49	100	100	100	100
Non-EMS transitions †	11,069	7,106	5,647	4,825	9,070
% Estimated error	0.24	0.17	0.15	0.14	0.21%
Eliminated RH SNPs	82,427	43,326	40,154	37,599	40,823
% RH	1.74%	1.04%	1.05%	1.10%	0.93%
Non- mutagenized Kronos	102	46	29	27	0
% EMS	39.2	60.9	82.8	85.2	na

* EMS-type mutations = G>A and C>T, excluding residual heterogeneity (RH).

† Reciprocal transitions A>G and T>C were used to estimate the error rate in EMS mutations.

Table S2. Correlations between promoter and exome capture for number of mutations and percent EMS and reciprocal non-EMS transitions across 1,492 common mutant lines at HetMC5/HomMC3 and 1,465 common mutant lines at EMS98%. Correlations were calculated using values obtained with both the previous global threshold HetMC5/HomMC3 for the exome capture remapped to CS RefSeq v1.1 and the new threshold method adjusted by library (EMS98%).

Trait	HetMC5/HomMC3 *R* (n = 1,492)	*P* value	EMS98% *R* (n = 1,465)	*P* value
No. EMS	0.854	***	0.881	***
%EMS (G>A/C>T)	0.154	***	0.034	ns
% Reciprocal A>G/T>C	0.183	***	0.119	***

Table S3. Comparison of SNP effects between previous (1) and new exome capture results. The new results are based on mapping the reads to the CS RefSeq v1.1 reference and using the EMS98% stringency method to call mutations. Annotation of SNP effects was performed using VEP from ENSEMBL (Method S3).

	Krasileva et al. (2017)	New
Number of Unique EMS mutations *	4,152,707	4,690,454
% EMS	99.10%	98.80%
Non-EMS-type transitions (estimated error) †	0.18%	0.34%
Missense variant	1,030,287	1,637,961
Splice acceptor variant	14,624	20,553
Splice donor variant	15,074	20,322
Stop gained	46,580	73,005
Synonymous variant	550,556	921,101
Gene models with at least one mutation (GM1)	48,172	57,950
GM1 with at least one truncation (splice and stop gain)	28,604	38,592
GM1 with at least one missense mutation	46,198	56,792
Avg. number of missense mutations per GM1	21.4	28.3
GM1 with truncation and/or deleterious missense ‡	43,787	55,303

* EMS-type mutations = G>A and C>T SNPs, excluding residual heterogeneity SNPs

† A>G and T>C SNPs

‡ A SIFT score < 0.05 was used to classify predicted deleterious missense mutations.

Table S4. Shared SNPs between promoter capture and exome capture.

SNP characteristics	Number of lines	Total SNPs
Promoter Capture	1,513	4,345,625
Exome Capture	1,521	4,748,394
Shared	1,465	174,658
% of Promoter capture	96.8%	4.0%
% of Exome capture	96.3%	3.7%

Table S5. Number of genes and EMS-type SNPs in the promoter and re-mapped exome capture per chromosome.

**Chromosome**	**HC Gene No. CS RefSeq. v1.1**	**Promoter captures**	**New exome capture**
1A	4,344	212,380	284,009
1B	4,714	220,188	303,578
2A	5,797	298,433	367,898
2B	6,109	313,699	403,738
3A	5,235	292,665	340,485
3B	5,905	294,564	372,226
4A	4,861	266,762	331,289
4B	3,869	219,342	273,502
5A	5,429	304,837	359,177
5B	5,576	326,000	388,667
6A	4,127	224,092	265,065
6B	4,615	231,492	295,899
7A	5,552	280,522	340,698
7B	4,855	257,069	309,623
chrUn	2,691	31,807	54,600
Kronos contigs *	Not available	513,509	Not available
**Total**	73,679	4,287,361	4,690,454

* The Kronos contigs were used only in the Promoter capture to identify SNPs and were also considered in the sequencing space.

Correlation	HC Gene No. CS RefSeq. v1.1	Promoter capture
Promoter captures	0.926	-
New exome capture	0.942	0.986

Table S6. Estimated average percent of GC sites accessible to EMS mutagenesis and proportion of these sites with at least one EMS-type mutation (saturation), using a Poisson approximation.

Trait	Promoter capture	Exome capture
Capture sequencing space	102,378,005	127,794,407
% GC in capture sequencing space	46.8%	47.3%
Total GC sites	47,861,717	60,446,755
Total EMS mutations	4,287,361	4,690,454
Mutated sites	3,914,089	4,343,084
λ that optimizes Poisson distribution	0.182	0.153
Mut. sites in 1 mutant line (Obs. / Exp.)	3,585,368 / 3,573,950	4,032,063 / 4,025,018
Mut. sites in 2 mutant lines (Obs. / Exp.) *	291,577 / 325,229	281,686 / 307,914
Mut. sites in 3 mutant lines (Obs. / Exp.)	31,539 / 19,731	25,214 / 15,704
Mut. sites in 4 mutant lines (Obs. / Exp.)	4,503 / 898	3,228 / 601
Estimated No. of GC sites without mutations	19,642,840	26,313,478
Estimated EMS-accessible GC sites	23,556,929	30,656,562
Estimated % EMS-accessible GC sites	49.2%	50.7%
Estimated % saturation EMS-accessible GC sites †	16.6%	14.2%
% duplicated mutations / total EMS mutations ‡	16.4%	14.0%

* The reduced number of observed mutations relative to the expected from Poisson distribution is likely affected by the MAPS pipeline, which eliminates mutations present in >1 line within the 48 lines included in one sequencing batch to eliminate potential homoeologous SNPs.

† Mutated sites / Estimated EMS-accessible GC sites.

‡ The % saturation estimated from the Poisson adjustment was validated by dividing the number of duplicated mutations present in >1 line by the total number of EMS mutations, which provides a similar probability.

Table S7. Effect of a mutation in a conserved SPL binding site on *VRN-A1* expression during spike development. The mutation in the promoter of the *VRN-A1* gene was identified in mutant line K4679. Expression was evaluated in young spikes (5 mm long) in sister lines with and without the mutation. Transcript levels were estimated by qRT-PCR relative to *ACTIN,* which was used as internal control. *VRN-A1* specific qRT-PCR primers were from a previously published study (6). Transcript levels are expressed as fold-*ACTIN* using the ΔCt method.

	Exp. 1 (BC_1F_2)		Exp. 2 (BC_1F_3)	
Sample	*SPL*-binding site mutant	WT sister line	*SPL*-binding site mutant	WT sister line
1	8.00	8.41	12.71	6.64
2	9.29	7.62	12.25	7.65
3	9.72	6.28	10.87	12.13
4	11.19	6.86	14.92	9.82
5	10.27	8.60	.	.
Mean	9.70	7.55	12.69	9.06
N	5	5	4	4
S.E.M	0.53	0.44	0.84	1.22
t-test	0.0145		0.0501	

ANOVA combining the two experiments and using experiment as block

***VRN1* transcript levels** relative to *ACTIN* (ΔCt method)

Source	DF	Sum of Squares	Mean Square	F Value	Pr > F
Model	2	57.8030	28.9015	11.31	0.0010
Error	15	38.3240	2.5549		
Corrected Total	17	96.1270			

R-Square: 0.601319

Source	DF	Type III SS	Mean Square	F Value	Pr > F
Exp	1	22.4950	22.4950	8.80	0.0096
Genotype	1	35.3080	35.3080	13.82	**0.0021**

Normality of residuals: Shapiro-Wilk test Pr < W = 0.9124

Least Squares Means

Genotype	qRT-PCR LSMEAN	Standard Error
WT	8.348	0.534
mut	11.149	0.534

VRN-A1 transcript levels in the SPL-binding site mutant were 33.6 % higher than in the wildtype sister line.

Table S8. Phenotypic effect of a mutation within a conserved SPL-binding site within the *VRN-A1* promoter. The mutation was identified in line K4679, and its effect on days to heading (DTH) and spikelet number per spike (SNS, avg. two taller tillers) was evaluated in two experiments.

	Exp. 1 DTH		Exp. 2 DTH		Exp. 1 SNS		Exp. 2 SNS	
Sample	*SPL*-bind. site mut.	WT sister line	SPL-bind. site mut.	WT sister line	SPL-bind. site mut.	WT sister line	SPL-bind. site mut.	WT sister line
1	46	45	48	51	16.0	16.0	16.0	15.5
2	45	45	53	49	15.5	14.0	15.0	15.0
3	46	44	46	52	16.0	15.5	14.5	16.0
4	44	44	52	50	15.0	13.5	16.0	15.0
5	45	45	50	50	16.0	15.5	16.0	16.0
6	.	.	49	48	.	.	15.0	14.5
7	.	.	49	46	.	.	16.0	14.0
8	.	.	46	48	.	.	14.0	15.5
Mean	45.20	44.60	49.13	49.25	15.70	14.90	15.31	15.19
S.E.M	0.37	0.24	0.90	0.67	0.20	0.48	0.28	0.25
t-tests	0.2165		0.9128		0.1656		0.7448	

ANOVAs combining the two experiments and using experiment as block

Days to heading

```
Source                  DF     S. Squares     Mean Square    F Value    Pr > F
Model                    2      113.27788        56.63894      17.33    <.0001
Error                   23       75.18365         3.26885
Corrected Total         25      188.46154
R-Square: 0.601066

Source                  DF    Type III SS     Mean Square    F Value    Pr > F
Exp                      1      113.12404       113.12404      34.61    <.0001
Genotype                 1        0.15385         0.15385       0.05    0.8302

Normality of residuals: Shapiro-Wilk test: Pr < W = 0.3098

Least Squares Means
                                Standard
Genotype      DTH LSMEAN           Error
WT              46.96683         0.50845
mut             47.12067         0.50845
```

Spikelet number per spike (power transformation **10 to restore Normality)

```
                                     Sum of
Source                      DF      Squares     Mean Square    F Value    Pr > F
Model                        2     1.78E23       8.903E22        0.86     0.4350
Error                       23     2.378E24      1.031E23
Corrected Total             25     2.554E24

R-Square: 0.069828

Source                      DF  Type III SS     Mean Square    F Value    Pr > F
Exp                          1     1.116E22        1.116E22       0.11     0.7452
Genotype                     1     1.669E23        1.669E23       1.62     0.2160

Normality of residuals: Shapiro-Wilk test: Pr < W = 0.0511

Least Squares Means (original scale not transformed)
                                Standard
Genotype      SNS LSMEAN           Error
WT            15.0826923       0.2204898
mut           15.4673077       0.2204898
Mutations in the putative SPL binding site were not associated with significant
differences in DTH and SNS
```

Table S9. Effect of a mutation in a conserved LFY-binding site on *VRN-A1* expression during spike development (Waddington 3.25). The induced EMS mutation was identified in line K2944 in the *VRN-A1* promoter. Transcript levels were estimated by qRT-PCR relative to *ACTIN,* which was used as internal control. *VRN-A1* specific qRT-PCR primers were from a previously published study (6). Transcript levels are expressed as fold-*ACTIN* using the ΔCt method.

Sample	Exp. 1 *VRN-A1* expression		Exp. 2 *VRN-A1* expression	
	LFY-binding site mut.	WT sister line	*LFY*-binding site mut.	WT sister line
1	1.52	3.29	6.83	5.13
2	3.73	1.36	5.72	4.56
3	3.86	1.39	5.57	5.81
4	5.72	2.36	5.48	5.39
5	.	.	6.11	5.00
6	.	.	4.99	5.48
7	.	.	5.67	5.12
8	.	.	6.41	4.70
Mean	3.71	2.10	5.85	5.15
S.E.M	0.86	0.46	0.20	0.14
t-test	0.1499		0.0143	

***VRN1* transcript levels** relative to *ACTIN* (ΔCt method). Experiment was used as block.

Source	DF	Sum of Squares	Mean Square	F Value	Pr > F
Model	2	41.9175	20.9588	27.48	<.0001
Error	21	16.0178	0.7628		
Corrected Total	23	57.9353			

R-Square: 0.6312

Source	DF	Type III SS	Mean Square	F Value	Pr > F
Exp	1	35.8975	35.8975	47.06	<.0001
Genotype	1	6.0200	6.0200	7.89	0.0105

Normality of residuals. Shapiro-Wilk test Pr < W = 0.3612

Least Squares Means

Genotype	Vrn1 LSMEAN	Standard Error
WT	3.7001	0.2599
M	4.7018	0.2599

VRN-A1 transcript levels in the LFY-binding site mutant are on average 27.1% higher than in the wildtype sister line.

Table S10. Phenotypic effects of the K2944 mutation in a conserved LFY-binding site within the *VRN-A1* promoter. The effect on spikelet number per spike (SNS) in the main shoot was evaluated in two independent experiments using BC_1F_2 and BC_1F_3 plants (derived from a single heterozygous BC_1F_2 plant). The effect on days to heading (DTH) and leaf number (LN) was evaluated in one experiment using BC_1F_3 plants.

Spikelet number per spike

	Exp. 1. Spikelet No. / spike			Exp. 2. Spikelet No. / spike	
	mutant	WT		mutant	WT
	13	15	14	15	16
	14	14	14	15	16
	12	14	15	15	15
	14	14	14	16	16
	15	15		14	17
	12	14		17	17
	13	14		14	17
	13	14			16
	12	14			17
	14	14			17
		13			16
		14			15
Average	13.2	14.1		15.1	16.3
n	10	16		7	12
S.E.M	0.33	0.13		0.40	0.22
t-test	0.0046			0.0168	
Kruskal-Wallis [1]	0.0113			not necessary	

[1] In Exp 1 no transformation restored normality of residuals so we used a non-parametric test

Average spikelet number per spike (SNS)

```
Data transformed using a power transformation (**-2) to improve normality of residuals.
For the untransformed data the P values for Genotype was 0.0002.

                                            Sum of
Source                         DF          Squares       Mean Square      F Value     Pr > F
Model                           2        0.0000242        0.0000121        39.67      <.0001
Error                          42        0.0000128        0.0000031
Corrected Total                44        0.0000370
R-Square: 0.65386

Source                         DF      Type III SS       Mean Square      F Value     Pr > F
Experiment                      1        0.0000184        0.0000184        59.00      <.0001
Genotype                        1        0.0000055        0.0000055        17.99      0.0001

Normality of residuals. Shapiro-Wilk test Pr < W       0.0129

Least Squares Means (untransformed)
                           Standard
Genotype      SNS LSMEAN      Error
M              14.1815       0.1940 6.6% decrease
WT             15.1826       0.1512
```

Leaf number and days to heading

	Exp. 2. Leaf Number		Exp. 2. Days to heading	
	mutant	WT	mutant	WT
	8	8	46	45
	9	9	46	46
	9	9	46	46
	9	8	47	46
	8	9	46	46
	9	9	47	46
	9	10	46	49
		9		47
		9		46
		9		47
		8		46
		9		45
Average	8.7	8.8	46.3	46.3
n	7	12	7	12
S.E.M	0.18	0.16	0.18	0.30
t-test	0.6533		0.9343	
Kruskal-Wallis [1]	0.6780		0.5888	

The ANOVAs using the parametric analyses was not significant for both leaf number and days to heading (*t*-Test above) but the residuals were not normally distributed, so we performed non-parametric Kruskal-Wallis test, which were also not significant:

Leaf number

```
Kruskal-Wallis Test
Chi-Square              0.1723
DF                           1
Pr > Chi-Square         0.6780
```

Days to heading

```
Kruskal-Wallis Test
Chi-Square              0.2922
DF                           1
Pr > Chi-Square         0.5888
```

Table S11. Relative abundance and % of heterozygous mutations among non-EMS errors.

	Exome Capture				Promoter Capture			
Type	Het	Hom	% Het	% of errors	Het	Hom	% Het	% of errors
**EMS mutations**								
CT	1,530,272	816,553	65.21		1,390,714	756,335	64.77	
GA	1,521,660	821,969	64.93		1,382,114	758,198	64.58	
% het. EMS mut.	3,051,932	1,638,522	65.07		2,772,828	1,514,533	64.67	
**Non-EMS errors**								
AC	2,097	0	100.0	3.6	4,070	1	100.0	7.0
TG	2,026	0	100.0	3.5	3,282	0	100.0	5.6
AG	7,756	1	100.0	13.4	4,539	0	100.0	7.8
TC	7,847	1	100.0	13.5	4,528	3	99.9	7.8
AT	5,849	0	100.0	10.1	5,528	0	100.0	9.5
TA	5,937	0	100.0	10.2	5,457	1	100.0	9.4
CG	2,518	0	100.0	4.3	1,468	1	99.9	2.5
GC	2,591	0	100.0	4.5	1,457	0	100.0	2.5
CA	10,660	0	100.0	**18.4**	13,564	1	100.0	**23.3**
GT	10,655	2	100.0	**18.4**	14,364	0	100.0	**24.7**
% het. non-EMS mut.	57,936	4	99.99		58,257	7	99.99	

SI References

1. K. V. Krasileva *et al.*, Uncovering hidden variation in polyploid wheat. *Proc. Natl. Acad. Sci. USA* **114**, E913-E921 (2017).
2. L. J. Gardiner *et al.*, Integrating genomic resources to present full gene and putative promoter capture probe sets for bread wheat. *Gigascience* **8**, giz018 (2019).
3. International Wheat Genome Sequencing Consortium, Shifting the limits in wheat research and breeding using a fully annotated reference genome. *Science* **361**, eaar7191 (2018).
4. P. C. Ng, S. Henikoff, SIFT: Predicting amino acid changes that affect protein function. *Nucleic Acids Res.* **31**, 3812-3814 (2003).
5. W. McLaren *et al.*, The Ensembl Variant Effect Predictor. *Genome Biol.* **17**, 122 (2016).
6. A. Loukoianov, L. Yan, A. Blechl, A. Sanchez, J. Dubcovsky, Regulation of *VRN-1* vernalization genes in normal and transgenic polyploid wheat. *Plant Physiol.* **138**, 2364-2373 (2005).
